# In silico screening for identification of novel β-1,3-glucan synthase inhibitors using pharmacophore and 3D-QSAR methodologies

**DOI:** 10.1186/s40064-016-2589-3

**Published:** 2016-07-04

**Authors:** Potshangbam Angamba Meetei, R. .S. Rathore, N. Prakash Prabhu, Vaibhav Vindal

**Affiliations:** Department of Biotechnology and Bioinformatics, School of Life Sciences, University of Hyderabad, Hyderabad, 500046 India; Bioinformatics Infrastructure Facility, School of Life Sciences, University of Hyderabad, Hyderabad, 500046 India; Centre for Biological Sciences, School of Earth, Biological and Environmental Sciences, Central University of South Bihar, Patna, 800014 India

**Keywords:** β-1,3-Glucan synthase, Pharmacophore, 3D-QSAR, Virtual screening, ADME

## Abstract

The enzyme β-1,3-glucan synthase, which catalyzes the synthesis of β-1,3-glucan, an essential and unique structural component of the fungal cell wall, has been considered as a promising target for the development of less toxic anti-fungal agents. In this study, a robust pharmacophore model was developed and structure activity relationship analysis of 42 pyridazinone derivatives as β-1,3-glucan synthase inhibitors were carried out. A five-point pharmacophore model, consisting of two aromatic rings (R) and three hydrogen bond acceptors (A) was generated. Pharmacophore based 3D-QSAR model was developed for the same reported data sets. The generated 3D-QSAR model yielded a significant correlation coefficient value (*R*^2^ = 0.954) along with good predictive power confirmed by the high value of cross-validated correlation coefficient (*Q*^2^ = 0.827). Further, the pharmacophore model was employed as a 3D search query to screen small molecules database retrieved from ZINC to select new scaffolds. Finally, ADME studies revealed the pharmacokinetic efficiency of these compounds.

**Electronic supplementary material:**

The online version of this article (doi:10.1186/s40064-016-2589-3) contains supplementary material, which is available to authorized users.

## Background

Invasive fungal infections have emerged as the major causes of morbidity and mortality associated with cancer chemotherapy, organ transplant, HIV infection, hematopoietic stem cell transplant, and intensive care hospitalization (Walsh et al. 1992; Groll et al. 1996; Minamoto and Rosenberg 1997; Sahbudak et al. 2015; Kanamori et al. 2015; Cabezas-Quintario et al. 2016; Kennedy et al. 2016). At present, only three structural classes of drugs: polyenes, azoles, and echinocandins are available as therapeutic options for such infections (Roemer and Krysan 2014). Among these, the polyenes like amphotericin B are the oldest class of antifungal drugs, which are preferred for treating systemic infections, whereas the most widely used class of antifungal drugs are Azoles (Maertens and Boogaerts 2000; Della Pepa et al. 2016). The echinocandins are the most recent addition to the available range of antifungal agents (Kitamura 2010; Matejuk et al. 2010; Moudgal and Sobel 2010). These compounds have emerged as an important therapeutic option against candidiasis as they exhibit broad fungicidal activity against *Candida* spp. (Roemer and Krysan 2014).

However, the major drawbacks seem to be in the usage of standard antifungal therapies, which includes toxicity, low efficacy rates and the development of resistance to these drugs, owing to their extensive consumption in recent years (Baixench et al. 2007; Chakrabarti et al. 2009; Howard et al. 2009; Pfaller et al. 2010). Therefore, to combat the problem of rapid increase in life-threatening fungal infections by drug-resistant fungal strains, there is a constant demand for the development of novel antifungal agents with a different mode of actions. The crucial point in the development of new drugs is the identification and characterization of new targets. The available antifungal drug targets with various underlying biological mechanisms are: ergosterol synthesis, chitin synthesis, glucan synthesis, nucleic acid synthesis, protein synthesis, and microtubules synthesis (Carrillo-Muñoz et al. 2006). Among them, glucan synthesis is one of the most important mechanisms, which leads to the synthesis of β-1,3-glucan, a major component of the fungal cell wall. The process is aided by the enzyme β-1,3-glucan synthase, which catalyzes the synthesis of β-1,3-glucan polymers (Cabib et al. 1982). The major function of the fungal cell wall is to control the internal turgor pressure, and its dynamics are closely related to the growth and division of the cell. Hence, any disturbance with its assembly or integrity ultimately leads to cell lysis and cell death. Since β-1,3-glucan synthase is unique to fungi and its activity is essential for cell wall assembly and cell growth, the enzyme has been considered as a promising target for the development of less toxic anti-fungal agents (Kurtz and Rex 2001). Indeed, echinocandins target the fungal cell wall by competitively inhibiting the synthesis of glucan polymers by binding the glucan synthase complex leading to the death of the fungal cell by damaging the cell wall (Cassone et al. 1981). The mode of action of echinocandins perhaps makes this particular class of compounds one of the most effective antifungal agents with minimal collateral toxicity in mammalian cells (Reboli et al. 2007).

One of the major limitations posed by echinocandins is that they could only be administered intravenously (Tattevin et al. 2014). Although some azoles like voriconazole and posaconazole could be administered orally but these antifungal agents exhibited higher a risk of drug interactions and increased pharmacokinetic variability (Boyd et al. 2004; den Hollander et al. 2006). Therefore, such limitations have led to the search for an orally bioavailable small molecule inhibitor that is highly desirable for antifungal treatment.

In recent years, pharmacophore based virtual screening is extensively being used to develop novel drugs (Shah et al. 2010; Yang 2010). A pharmacophore often serves as a template for the desired ligand in search of a potential drug (Van Drie 1997; Güner 2000; Yang 2010). In the present study, pharmacophore models were developed using PHASE (Dixon et al. 2006) [24] to explore new lead compounds. Further, based on the alignment of the pharmacophoric points, an atom-based 3D-QSAR model was generated. The pharmacophore model was employed to screen ZINC database of synthetic and natural product molecules (Irwin et al. 2012). The contour maps produced from 3D-QSAR studies elucidated the essential structural features required for glucan synthase (GS) inhibition that could be used as a guideline for the further design of more potent inhibitors.

## Methods

### Dataset

A dataset of 42 molecules containing pyridazinone derivatives of *Candida albicans* β-1,3-glucan synthase inhibitors were used for the pharmacophore based 3D-QSAR studies. All the molecules with their inhibitory activities were collected from the literature (Ting et al. 2011; Zhou et al. 2011). The GS inhibitory activities expressed in terms of IC_50_ were then converted to the pIC_50_ (−log IC_50_). For selecting the training and test set molecules, careful consideration was taken to include diverse molecules as well as an optimum proportion of active and less active molecules for the uniform sampling of data. Out of the 42 molecules, ~73 % i.e., 31 were used as the training set for the generation of 3D-QSAR models and the rest ~27 % were used as the test set for external validation (Table 1). The detail procedure for selecting the training and test sets molecules is described in Additional file 1. The pIC50 values for training set spanned over 3 log units. Further, to develop the pharmacophore models, the molecules were assigned as actives (pIC_50_ > 6.500) and inactives (pIC_50_ < 5.640) as given in Table 1.

**Table 1:** Training and test set molecules with their observed and predicted activities


Compound	R	pIC_50_ observed	pIC_50_ Predicted	Fitness score^c^	Set
1		6.39	6.38	2.74	Test
2		6.20	6.30	2.9	Test
3		6.37	6.31	2.92	Training
4		6.38	6.33	2.56	Training
5^a^		7.13	7.07	2.72	Training
6^a^		6.79	9.94	2.71	Test
7^a^		6.66	6.61	3	Training
8		6.09	6.37	2.79	Training
9^a^		6.54	6.61	2.81	Training
10^a^		7.10	7.06	2.85	Training
11^b^		4.64	4.53	2.06	Training


Compound	R	pIC_50_ observed	pIC_50_ predicted	Fitness score	Set
12^a^		6.70	6.74	2.95	Training
13	–CH3	5.66	5.95	2.23	Test
14		5.89	5.97	2.78	Training
15		5.77	5.99	2.85	Training


Compound	R	pIC_50_ observed	pIC_50_ predicted	Fitness score	Set
16^b^		5.33	5.18	2.08	Test
17		5.65	5.64	2.33	Training
18^a^		6.74	6.43	2.95	Training
19		5.64	5.62	0.81	Training
20		5.82	5.50	2.26	Test
21^a^		6.92	7.04	2.76	Training
22		5.96	6.04	2.31	Training
23^b^		5.52	5.46	2.21	Training
24		6.27	5.87	2.34	Test
25		6.04	6.00	2.22	Test


Compound	R	pIC_50_ observed	pIC_50_ predicted	Fitness score	Set
26	H	6.04	6.09	2.11	Training
27^b^		5.38	5.38	2.7	Training


Compound	R	R1	pIC_50_ observed	pIC_50_ predicted	Fitness score	Set
28		H	6.38	6.21	2.79	Training
29		H	5.96	6.16	2.78	Training
30		F	6.21	6.20	2.78	Test
31		F	6.22	6.14	2.78	Training


Compound	R	pIC_50_ observed	pIC_50_ predicted	Fitness score	Set
32		5.66	5.71	2.52	Training
33^b^		5.55	5.56	2.81	Training


Compound	R	pIC_50_ observed	pIC_50_ predicted	Fitness score	Set
34^b^		5.05	5.09	2.33	Training
35^b^		5.21	5.31	2.84	Training
36		6.00	6.16	2.87	Test
37		6.28	6.33	2.87	Training
38		6.21	6.14	2.86	Training
39^a^		6.6	6.32	2.86	Test
40		6.58	6.32	2.86	Training
41		6.04	5.86	2.87	Training
42		5.72	5.83	2.87	Training

^a^Active pharm set

^b^Inactive pharm set

^c^Fitness score measures the alignment of the pharmacophore site points of matching compounds to those of the hypothesis. The reference ligand for the hypothesis having an exact match has a perfect fitness score of 3.0

Indicates the position for R substitution

### Ligand preparation

2D structures were drawn using 2D sketcher of Maestro (Maestro 2013), the 3D structure conversion and minimizations of ligands were performed using LigPrep (LigPrep 2013). Various possible ionization states were generated at pH 7 ± 2.0. In order to develop a pharmacophore model, 3D realistic representations of the experimental molecular structures are necessary. Since most ligands were flexible, it is important to consider a range of thermally accessible conformational states to increase the chances of finding geometry close to the putative binding mode. PHASE provides two built-in approaches, both of which employ the MacroModel conformational search engine. Conformers were generated using ConfGen (Watts et al. 2010) followed by minimization of each generated structure using Merck Molecular Force Field (MMFF).

### Common pharmacophore hypothesis (CPH) generation

The chemical features or the pharmacophore sites for the molecules were defined using built-in six pharmacophoric features (Dixon et al. 2006) hydrogen-bond acceptor (A), hydrogen-bond donor (D), hydrophobic group (H), negatively charged group (N), positively charged group (P), and aromatic ring (R). The common pharmacophore hypothesis was identified by using the active analog approach, in which common pharmacophores were collected from the conformations of the set of active ligands using a tree-based partitioning technique that groups together similar pharmacophores according to their inter-site distances. The tree depth was given as 5 with an initial box size of 32 Å and a final box size of 1 Å. Subsequently, the pharmacophores were scored and ranked to identify the best candidate hypothesis. The scoring algorithm included the contributions from the alignment of site points, vectors, volume overlap, selectivity, number of ligands matched, relative conformational energy and activity.

### 3D-QSAR model generation and validation

The pharmacophoric points of the best CPH was used for the alignment of the molecules, included in the 3D-QSAR studies. A rectangular grid is defined to encompass the space occupied by the aligned ligands. This grid divides the occupied space into N uniformly sized cubes, typically 1 Å on each side. Subsequently, the biological activity values (pIC_50_) were correlated to the best hypothesis to generate a 3D-QSAR model, which mapped necessary structural features of molecules that govern activity. The model was generated with 1 Å grid spacing and a maximum number of PLS factors of 4 by choosing the atom based 3D-QSAR option. The best 3D-QSAR model was selected on the basis of the statistical robustness and validated by predicting the activity of the external test set molecules. External validation is a crucial aspect of pharmacophore design as it is considered as a definitive proof for judging predictability of a model, particularly when the model is built for the purpose of predicting activities of compounds in an external test set (Boyd et al. 2004). The external predictability of the selected model was determined by using the standard deviation of error prediction $R_{pred}^{2}$ (Roy and Roy 2008) for the test set calculated according to the following equation:

$$R_{pred}^{2} = 1 - \frac{{\sum \left( {Y_{{pred\left( {Test} \right) - }} Y_{Test} } \right)^{2} }}{{\sum \left( {Y_{Test - } \bar{Y}_{Training} } \right)^{2} }}$$

where, *Y*_*pred* (*Test*)_ and *Y*_*Test*_ indicate predicted and observed activity values of test set compounds respectively, and $\bar{Y}$_*Training*_ represent mean activity value of the training set molecules.

### Virtual screening

To investigate novel scaffolds with potential GS inhibitory activity, in silico screening of synthetic compounds (‘lead like’ compound consisting of 2,46,580 molecules) as well as natural product (41,490), available in ZINC database, was performed using the derived pharmacophore model. The resulting hits were filtered using Lipinski’s criteria (Lipinski et al. 1997) and then the compounds which have fitness score of more than 2 and 1.5 for synthetic and natural products, respectively, were subjected to ADME studies. The relaxed criterion for fitness score for the natural product was used to obtain a significant number of hits.

### ADME (absorption, distribution, metabolism, and excretion) studies

ADME properties were calculated using QikProp (2013). It calculates regression modeling-based 44 properties of physiochemical and pharmacokinetical relevance using the experimental results of over 710 compounds including about 500 drugs. Further, it also evaluates the acceptability of hits based on the Lipinski’s rule of five, provides ranges for comparing a particular molecule’s properties with those of 95 % of known drugs, and retrieve most similar drugs available.

## Results and discussion

### Pharmacophore modeling and 3D-QSAR studies

**Fig. 1:**
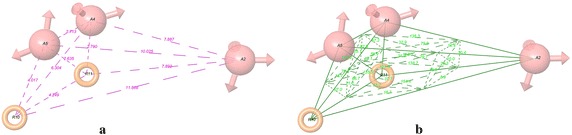
Pharmacophore hypothesis AAARR.594 characterized by three hydrogen bond acceptors and two aromatic rings. **a** Distances (in Å) and **b** angles (°) between different pharmacophoric features

The pharmacophore models were constructed by selecting a minimum of four and a maximum of five pharmacophoric sites. Consequently, from a list of 9 variant combinations a total of 5887 five-featured CPHs belonging to eight types, AAHRR, AHHRR, AAAAR, AAARR, AAAHH AAAAH, AAAHR, and AAHHR were subjected to rigorous scoring analysis with respect to actives using default parameters for site, vector, and volume. Reference relative conformational energy (kJ/mol) was incorporated in the score, and ligand activity, expressed as pIC_50_, was included with a default weight. Hypotheses generated from this process were subsequently scored with respect to the seven inactives, using a weight of 1.0. The best hypothesis AAARR.594 was selected for further study on the basis of the highest survival score (Table 2) as well as according to the best scoring statistical parameters generated from pharmacophore based 3D-QSAR models (Table 3). The AAARR.594 hypothesis consists of three hydrogen bond acceptors (A) and two aromatic rings (R) (Fig. 1). The distances and angles between different sites of AAARR.594 are given in Table 4.

**Table 2:** Score of different parameters of top 4 hypotheses

Hypothesis	Survival^a^	Survival-inactive^b^	Post-hoc^c^	Site^d^	Vector^e^	Volume^f^	Selectivity^g^	No. of matches^h^	Energy^i^	Activity^j^	Inactive^k^
*AAARR.594*	*3.827*	*1.288*	*3.827*	*0.98*	*0.999*	*0.844*	*1.509*	*9*	*0.124*	*6.66*	*2.539*
AAAAR.3017	3.826	1.287	3.826	0.98	0.999	0.844	1.370	9	0.124	6.66	2.540
AAAHR.2612	3.791	1.734	3.791	0.96	0.998	0.832	1.602	9	0.132	6.74	2.056
AAAAH.7206	3.790	1.453	3.79	0.96	0.998	0.832	1.402	9	0.132	6.74	2.338

Best hypothesis selected for further study is shown in italic

^a^Survival score: provides an overall ranking of the hypotheses and is calculated as: survival score = (Vector score) + (Site score) + (Volume score) + (Selectivity score) + (Number of actives that match the hypothesis − 1) − (Reference-ligand relative conformational energy) + (Reference-ligand activity)

^b^Survival-inactive: survival score for actives with a multiple of the survival score for inactives subtracted

^c^Post-hoc: result of rescoring and is the combination of active and inactive survival score

^d^Site score: This score measures how closely the site points are superimposed in an alignment to the pharmacophore of the structures that contribute to this hypothesis, based on the RMS deviation of the site points of a ligand from those of the reference ligand

^e^Vector score: measures how well the vectors for acceptors, donors, and aromatic rings are aligned in the structures that contribute to this hypothesis

^f^Volume score: It is the average of the individual volume scores. The individual volume score is the overlap of the volume of an aligned ligand with that of the reference ligand, divided by the total volume occupied by the two ligands

^g^Selectivity score: the fraction of molecules likely to match the hypothesis, regardless of their activity toward the receptor. Possible range is 0 upward. A score of 2 means 1 in 100 molecules would match the hypothesis. Higher the selectivity score, better is the selected hypothesis

^h^No. of matches: number of actives that match the hypothesis (9 actives in this case)

^i^Energy: relative energy of the reference ligand. The possible range is 0 upward. Energy of 0 kcal/mol means that the reference ligand is the lowest energy conformation

^j^Activity: activity of the reference ligand

^k^Inactive: this score is used as a penalty to the survival scores (number of total inactives included = 6). Lower value is better for hypothesis (minimum value can be 1 as minimum one inactive must be included in the hypothesis development)

**Table 3:** Summary of PHASE 3D-QSAR statistical results of the top four hypotheses, best model is shown in italic font

Hypothesis^a^	SD^b^	R-squared^c^	F^d^			P^e^		Stability^f^	RMSE^g^	Q-squared^h^	Pearson-R^i^	$R_{pred}^{2}$ ^j^
*AAARR.594*	*0.1368*	*0. 9542*	*135.30*			*5.277e* **−** *017*		*0.4923*	*0.1953*	*0.8268*	*0.9103*	*0.711*
AAAAR.3017	0.1458	0.9480	118.5			2.707e−160		0.2039	0.2342	0.7647	0.8112	0.678
AAAHR.2612	0.1455	0.9482	118.9			2.597e−16		0.2287	0.2517	0.6808	0.8023	0.634
AAAAH.7206	0.1365	0. 9530	131.8			7.3e−17		0.1528	0.2532	0.7627	0.8233	0.679

^a^Hypotheses used in the analyses

^b^(SD) the standard deviation of regression

^c^(*R* ^2^) coefficient of determination

^d^(F) the ratio of the model variance to the observed activity variance

^e^(P) significance level of F when treated as a ratio of Chi squared distributions

^f^(Stability) stability of the model predictions to changes in the training set composition

^g^(RMSE) the RMS error in the test set predictions

^h^(*Q* ^2^) directly analogous to *R* ^2^ but based on the test set predictions

^i^(Pearson-R) value for the correlation between the predicted and observed activity for the test set

^j^($R_{pred}^{2}$) Predictive *R* ^*2*^, Standard deviation of error prediction

**Table 4:** Distance and angles between different sites of AAARR.594

Site1	Site2	Distance (Å)	Site1	Site2	Site3	Angle
A2	A4	7.887	A4	A2	A5	10.8
A2	A5	10.025	A4	A2	R10	29.3
A2	R10	11.868	A4	A2	R11	20.4
A2	R11	7.893	A5	A2	R10	18.8
A4	A5	2.713	A5	A2	R11	10
A4	R10	6.304	R10	A2	R11	8.9
A4	R11	2.79	A2	A4	A5	136.3
A5	R10	4.017	A2	A4	R10	113
A5	R11	2.635	A2	A4	R11	79.9
R10	R11	4.249	A5	A4	R10	25.1
			A5	A4	R11	57.2
			R10	A4	R11	33.1
			A2	A5	A4	32.9
			A2	A5	R10	107.5
			A2	A5	R11	31.3
			A4	A5	R10	138.2
			A4	A5	R11	62.9
			R10	A5	R11	76.3
			A2	R10	A4	37.7
			A2	R10	A5	53.7
			A2	R10	R11	16.7
			A4	R10	A5	16.7
			A4	R10	R11	21
			A5	R10	R11	37
			A2	R11	A4	79.7
			A2	R11	A5	138.7
			A2	R11	R10	154.4
			A4	R11	A5	59.9
			A4	R11	R10	125.9
			A5	R11	R10	66.7

**Fig. 2:**
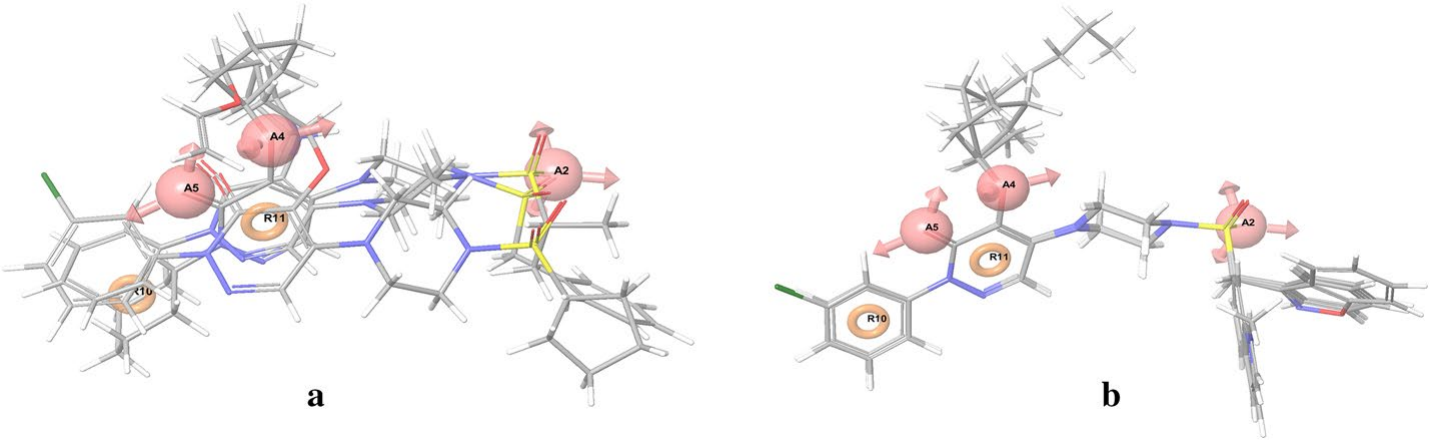
Pharmacophore AAARR.594 mapped on inactive ligands (**a**) and active ligands (**b**)

The generated pharmacophore should be able to differentiate between active and inactive molecules, to avoid unwanted molecules during virtual screening. To validate the pharmacophore model, it was mapped to inactive and active compounds (Fig. 2). A wrong hypothesis would show better mapping result with inactive molecules. However, from Fig. 2 it is evident that the pharmacophore model mapped well in case of active than the inactive molecules, thus justifying the pharmacophore model for using in screening.

**Fig. 3:**
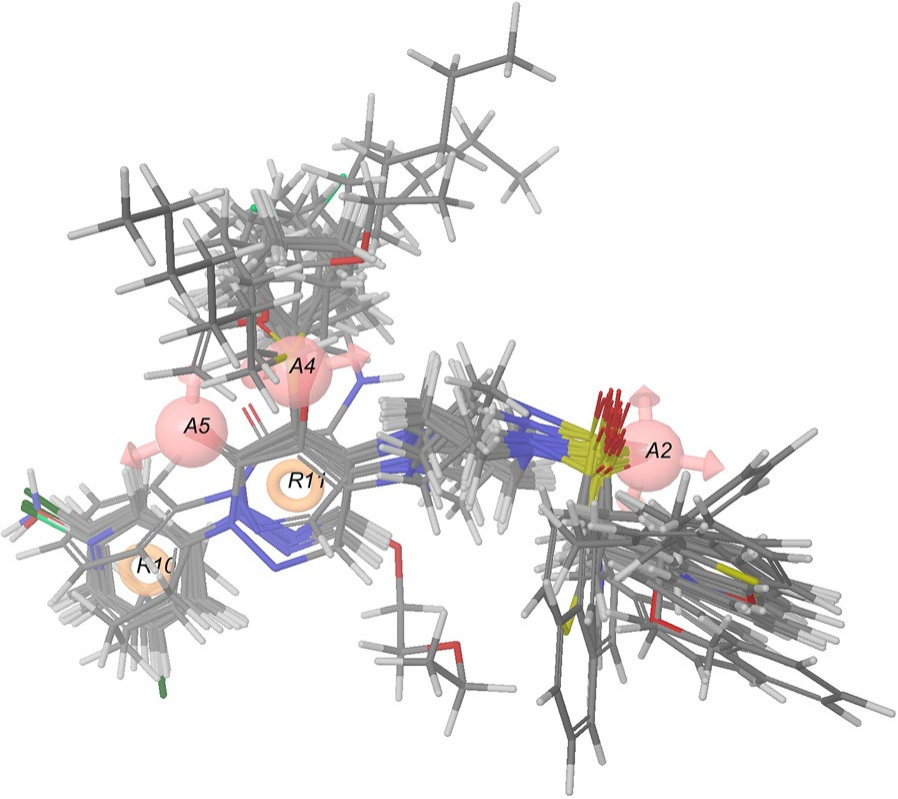
Alignment of the training set molecules on the best scoring pharmacophore AAARR.594

Further, for constructing the atom-based 3D-QSAR model of AAARR.594 hypothesis, the training set compounds were aligned on the pharmacophoric features (Fig. 3) and was explored by PLS analysis with one to four PLS factors. The predictive power of each model was evaluated by the test set compounds. A summary of the statistical data for 3D-QSAR analysis of AAARR.594 hypothesis is listed in Table 5. Different statistical parameters such as *R*^2^, *Q*^2^, SD, and RMSE were taken into consideration to examine the robustness of the 3D-QSAR models and the model with four PLS factors was found to be the best. The large value of *F* (*F* = 135.30) indicates a statistically significant regression model, which is also supported by the small value of statistical significance (*P* = 5.277^−17^), an indication of a high degree of confidence. Furthermore, the small value of standard deviation (*SD* = 0.1368) of the regression and root-mean-square error (*RMSE* = 0.1788) and the higher value of the QSAR model stability (stability = 0.4923) indicates that the data used for model generation is the best for the QSAR analysis. The model with PLS factor four has the highest *Q*^2^ (*Q*^2^ = 0.827) together with high Pearson-*R* value (Pearson-*R* = 0.9103) and *R*^2^ (*R*^2^ = 0.954), suggesting a close correspondence between predicted and actual IC_50_ activity values.

**Table 5:** Summary of PHASE 3D-QSAR statistical results of AAARR.594 hypothesis, best model is shown in italic font

No. of factors^a^	SD^b^	R-squared^c^	F^d^	P^e^	Stability^f^	RMSE^g^	Q-squared^h^	Pearson-R^i^	$R_{pred}^{2}$ ^j^
1	0.3265	0.7089	70.60	2.909e−009	0.7744	0.2226	0.7318	0.8101	0.656
2	0.2281	0.8628	88.10	8.336e−013	0.7296	0.2170	0.7450	0.8900	0.698
3	0.1613	0.9339	127.20	4.879e−016	0.5590	0.2015	0.7801	0.9011	0.689
*4*	*0.1368*	*0. 9542*	*135.30*	*5.277e*−*017*	*0.4923*	*0.1953*	*0.8268*	*0.9103*	*0.711*

^a^Number of factor used in the analyses

^b^(SD) the standard deviation of regression

^c^(*R* ^2^) coefficient of determination

^d^(F) the ratio of the model variance to the observed activity variance

^e^(P) significance level of F when treated as a ratio of Chi squared distributions

^f^(Stability) stability of the model predictions to changes in the training set composition

^g^(RMSE) the RMS error in the test set predictions

^h^(*Q* ^2^) directly analogous to *R* ^2^ but based on the test set predictions

^i^(Pearson-R) value for the correlation between the predicted and observed activity for the test set

^j^($R_{pred}^{2}$) Predictive *R* ^*2*^, Standard deviation of error prediction

Since the difference between *R*^2^ and *Q*^2^ is <0.3 (0.954–0.827 = 0.127) the model is considered to be good and reliable (Eriksson et al. 2003; Eriksson 2008). Moreover, the high $R_{pred}^{2}$ value (0.711) shows that the model has strong predictive power and significance.

**Fig. 4:**
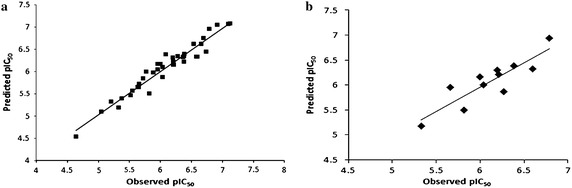
Fitness graph between observed activity v/s PHASE predicted activity of **a** training and **b** test set molecules

The plots for experimental against predicted activities of the training and test set ligands are given in Fig. 4. All the values for the training and test set molecules were plotted around the best fit line demonstrating the significance of the predicted model. Furthermore, the best fitted model was employed to predict the biological activities of training and test set molecules as shown in Table 1.

To check the superiority of the generated 3D-QSAR model based on the highest scoring pharmacophore hypothesis over the rest of the pharmacophore hypotheses, a 3D-QSAR model was developed from the least scoring pharmacophore hypothesis AAAHH.338. The statistical result of the generated 3D-QSAR model is given in Additional file 2: Table S1. The low statistical parameters such as *R*^2^ = 0.8684, *Q*^2^ = 0.5952, and $R_{pred}^{2}$ = 0.412 suggest that the 3D-QSAR model derived from AAARR.594 hypothesis could be the best for further studies.

### 3D-QSAR contour map analysis

**Fig. 5:**
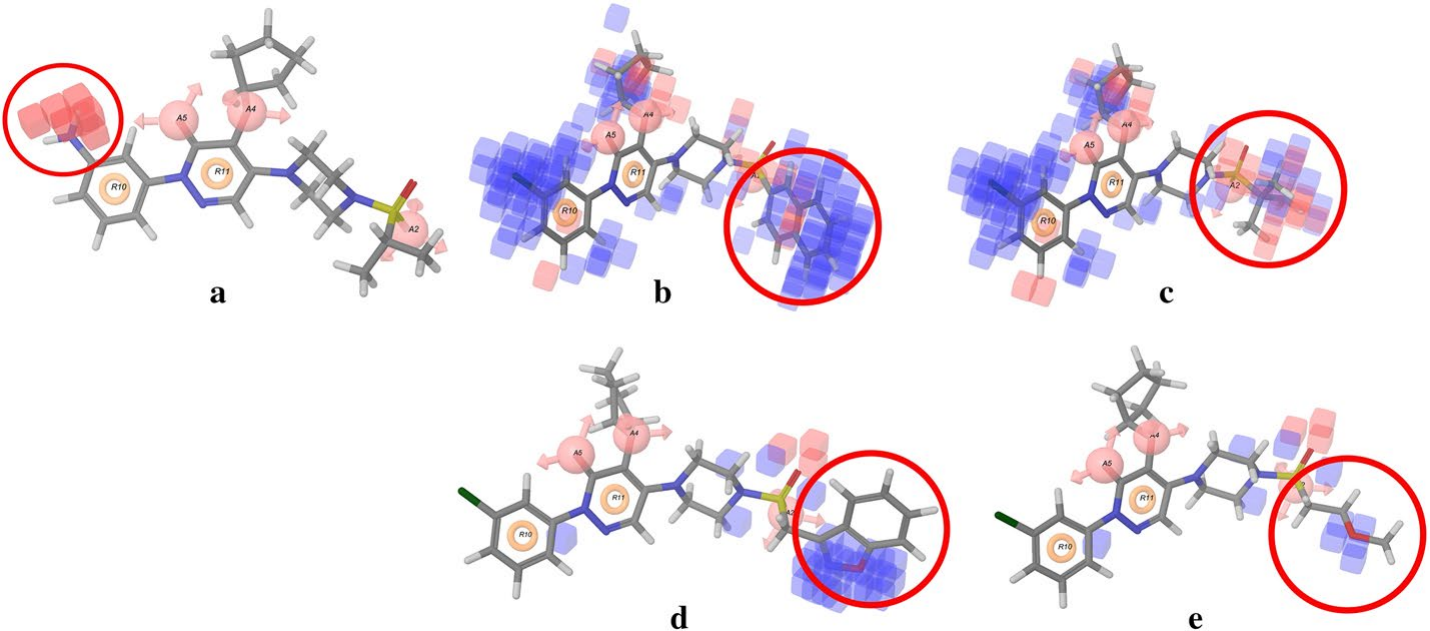
3D-QSAR contour map in the context of **a** Hydrogen bond donor represented on molecule 42; **b** hydrophobic interactions shown on molecule 5 and **c** 1; **d** Electron withdrawing contours overlapped on molecule 10 and **e** 3; *blue cubes* indicate favorable *while red cubes* unfavorable coefficients; *circles* signifies the substitution position under consideration for the contour map analysis

The atom-based 3D-QSAR results can be visualized as a cluster of cubes representing the pharmacophore regions, the blue cubes indicating favorable and the red ones unfavorable for activity. Specific structural features or functional groups, having blue and red cubes may increase or decrease activity, respectively. A pictorial representation of the contour maps representing hydrogen bond acceptor, hydrophobic and electron withdrawing properties for the best QSAR model is shown in Fig. 5.

### Hydrogen bond donor contour map analysis

The hydrogen bond donor contour map applied to the molecule **42** is shown in Fig. 5a. A cluster of red cubes can be seen on the NH2 group of the phenylamine substitution at the R10 pharmacophoric position. The red contour signifies unfavored region for activity and, therefore, the substitution with hydrogen bond donor group at this position reduced the GS inhibitory activity.

### Hydrophobic contour map analysis

As shown in Fig. 5b, a large blue contour map is observed near the sulfonamide tail of compound **5** which has naphthalene group substitution at the A2 position of the pharmacophoric feature and hence has higher activity. However in the case of molecule **1**, (Fig. 5c) the particular position has more red cubes and is substituted with a *tert*-butyl moiety, which is less hydrophobic, thus resulting in lower activity. Again at the A4 position of the pharmacophoric site, the pyridazinone ring has long acyclic chain substitutions with various linkers. Molecule **21** with linker oxygen is more active since the substituted moiety is enclosed in the favored blue contours, whereas molecule **23** projects its sulfonamide linker into the red contours (shown in Additional file 3: Figure S1a) and, therefore, has lower activity. Further, large blue contours can be seen at the R10 position of the pharmacophoric feature for compound **39**, the *m*-chlorofluorobenzene substitution is responsible for the higher activity, which overlapped with the blue contours. Conversely, in the case of molecule **34** the cyclohexyl moiety is less hydrophobic and it extends into the red contours, resulting in lower activity (Additional file 3: Figure S1b).

### Electron withdrawing contour map analysis

The favored region of electron withdrawing blue contoured map, covering the molecule **10** shown in Fig. 5d can be explained on the basis of the benzoxazolemethyl substitution on the sulfonamide tail at the A2 pharmacophoric position. The nitrogen and oxygen atoms present in the oxazole ring make the moiety more electronegative, which is favorable at this position and hence has higher activity. In contrast, the methoxyethane group of molecule **3** at A2 pharmacophoric position (Fig. 5d) is less electronegative and thus has lower activity as compared to molecule **10**. Additionally, the red contour map at the R10 pharmacophoric position makes the molecules with electronegative substitutions less active. For example, molecule **42** with aniline substitution at that position has low activity, which falls into the red contour map (Additional file 3: Figure S1c). Similarly, molecule **35** having pyridine substitution also has low activity.

### Virtual screening

**Fig. 6:**
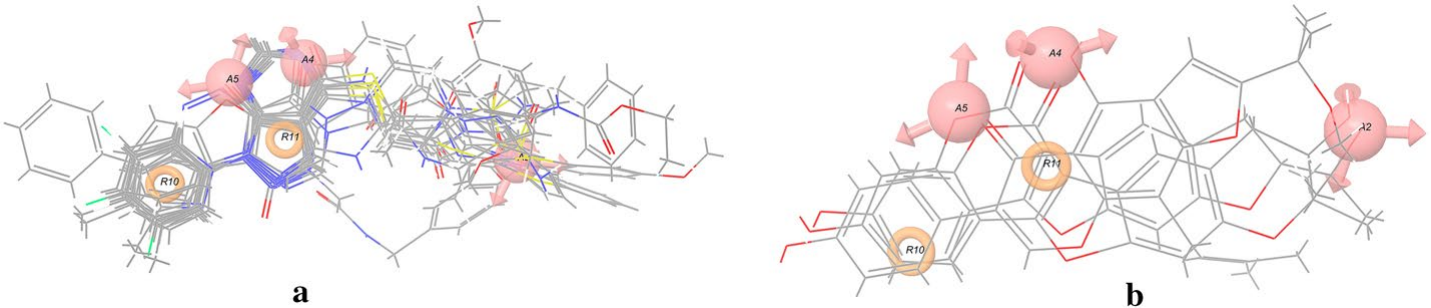
Lead molecules with a total of **a** 11 synthetic compounds and **b** 3 natural product hits retrieved from pharmacophore virtual screening shown on the five pharmacophoric features of AAARR.594 hypothesis

The selected hypothesis AAARR.594 was used to screen 2,46,580 synthetic and 41,490 natural product compounds available in ZINC database. For searching the database, the minimum criteria used was matching of all the five pharmacophore features, with default tolerance on matching the features to each of the five inter-feature distances. Each molecule of the database was represented by a maximum of 100 conformations and conformers were generated using ConfGen. A total of 672 compounds, consisting of 560 synthetic and 112 natural product hits, were screened, after filtering using Lipinski criteria. Additionally, to narrow down the number of potential hits, the matches were filtered using fitness score. Only those hits were selected, which had fitness score of more than 2 and 1.5 for synthetic and natural product compounds, respectively. A total of 11 synthetic compounds survived the process, and 3 hits were retrieved from the natural product database (Table 6). These lead molecules overlapped with the five pharmacophoric features as shown in Fig. 6.

**Table 6:** Lead molecules obtained from pharmacophore screening

S. no	ZINC database ID	Compound (synthetic)	Fitness score
1	ZINC40390784		2.13
2	ZINC57742232		2.12
3	ZINC09823898		2.10
4	ZINC09087176		2.09
5	ZINC21010902		2.08
6	ZINC18222209		2.06
7	ZINC81275318		2.05
8	ZINC03522823		2.05
9	ZINC12140774		2.03
10	ZINC69708581		2.00
11	ZINC72283844		2.00

S. no	ZINC database ID	Compound (natural product)	Fitness score
12	ZINC38269114		1.76
13	ZINC13375730		1.74
14	ZINC13375727		1.61

### ADME prediction

An analysis of 44 physicochemically important descriptors and pharmaceutically relevant properties of the 14 lead compounds were carried out using Qikprop. Only the significant descriptors including Lipinski’s, which are reported to be essential for predicting drug-like properties of molecules were considered (Table 7). They were: molecular weight in g/mol (acceptable range of 150–650), Octanol/water partition coefficient (QlogP o/w, −2 to 6.5), aqueous solubility (QPlogS in mol/L, −6 to 0.5), Caco-2 cell permeability (QPPCaco in nm/s, <25 poor and >500 high), Brain/blood partition coefficient (QPlogBB in ml blood/g brain, −3.0 to 1.2), apparent MDCK cell permeability (QPPMDCK in nm/s, <25 poor, higher the value of MDCK cell, higher is the cell permeability), Percent human oral absorption (>80 % is high, <25 % are poor). The partition coefficient (QPlogPo/w) and water solubility (QPlogS), which are critical for estimation of absorption and distribution of drugs within the body, ranged between 0.8 and 5.3 and −8.092 to −3.102, respectively. Cell permeability (QPPCaco), a key factor governing drug metabolism and its access to biological membranes ranged from 62.460 to 812.195. The predicted apparent MDCK cell permeability, QPPMDCK ranges from 24.691 to 2955.977 nm/s. Blood–brain partition coefficient (QlogBB) ranges from −3.0 to 1.2. The percentage human oral absorption of the compounds ranged from 66.7 to 100 %. The pharmacokinetic properties of the lead molecules are within the acceptable range, defined for human use hence indicating their potential as drug-like molecules.

**Table 7:** Calculated ADME properties of top 14 hits using QikProp

S. no	ZINC database ID	mol MW^a^	QPlogPo/w^b^	QPlogS^c^	QPPCaco^d^	QPlogBB^e^	QPPMDCK^f^	Percent human oral absorption^g^
1	ZINC40390784	491.508	4.344	−7.103	283.006	−1.661	126.417	96.262
2	ZINC57742232	349.425	1.579	−4.142	174.722	−1.624	111.303	76.327
3	ZINC09823898	473.505	4.047	−7.408	122.68	−2.058	73.068	88.025
4	ZINC09087176	420.485	4.658	−6.158	1697.891	−0.627	1250.849	100
5	ZINC21010902	405.449	2.26	−3.882	637.579	−0.766	549.709	90.375
6	ZINC18222209	406.476	2.96	−5.739	313.892	−1.142	345.347	88.966
7	ZINC81275318	314.404	3.999	−5.111	1361.266	−0.539	1067.441	100
8	ZINC03522823	342.375	1.668	−3.596	47.592	−1.645	57.928	66.738
9	ZINC12140774	487.443	3.321	−4.663	615.698	−0.608	764.772	96.313
10	ZINC69708581	333.355	2.197	−4.176	495.559	−0.878	231.624	88.048
11	ZINC72283844	341.366	1.384	−3.292	345.62	−1.083	234.06	80.484
12	ZINC38269114	434.488	5.388	−6.92	644.585	−1.148	307.755	95.814
13	ZINC13375730	352.343	2.666	−4.664	342.987	−1.094	155.611	87.933
14	ZINC13375727	352.343	1.54	−3.829	194.191	−1.313	84.141	76.916

^a^Predicted molecular weight in g/mol (mol_MW) (acceptable range: 150–650)

^b^Predicted Octanol/water partition coefficient (QlogP o/w) (acceptable range −2 to 6.5)

^c^Predicted aqueous solubility in mol/L (QPlogS) (acceptable range −6 to 0.5)

^d^Predicted Caco-2 cell permeability in nm/s (<25 is poor and >500 is high)

^e^Predicted brain/blood partition coefficient in ml blood/g brain (QPlogBB) (Acceptable range −3.0 to 1.2)

^f^Predicted apparent MDCK cell permeability in nm/s (QPPMDCK) (<25 poor)

^g^Percentage of human oral absorption (<25 % is poor and >80 % is high)

### Natural product hits

The lead molecules from the natural product database that survived the screening process are found to be present in the medicinal plants *Erythrina variegate* and *Psoralea corylifolia* (Meetei et al. 2014). The ZINC entry ZINC382691140 is an isoflavonoid, present in the stem bark of *Erythrina variegate* (Xiaoli et al. 2006). Entries ZINC13375727 and ZINC13375730 are found in the seed of *Psoralea corylifolia* (Gupta et al. 1990). These plants are used in the folklore system of medicine in various parts of the world. *Erythrina variegate* is used in various treatments such as fever, joint pains, skin diseases and also to relieve from earache and toothache. Additionally, the extract from the plant has shown significant antimicrobial activity (Rahman et al. 2007; Sahoo et al. 2012). Similarly, the medicinal plant *Psoralea corylifolia* is used in many diseases including various skin diseases, nephritis, cardiovascular diseases, cancer, and osteoporosis. The plant extract was shown to have antifungal, antibacterial, antitumor, antioxidant and anti-inflammatory activities (Chopra et al. 2013; Zhang et al. 2016).

The discovery of natural products from the pharmacophore screening process could be an alternative source for the detection of novel medicinal plant remedies with better safety and efficacy profiles.

### Validation by prediction of known inhibitors

Four known inhibitors of β-1,3-glucan synthase: enfumafungin, ergokonin A, arundifungin and ascosteroside were collected from literature (Onishi et al. 2000). The 2D to 3D conversion, and optimization of the molecules were done as described above (“Ligand preparation” section). The molecules were aligned on the generated pharmacophore (AAARR.594) and their biological activities were predicted using the 3D-QSAR model. The observed and predicted biological activities of the compounds are comparable (Additional file 2: Table S2) suggesting that this model has accurate predictability.

## Conclusion

The present report portrays different pharmacophore hypotheses of pyridazinone derivatives as inhibitors against glucan synthase. 3D-QSAR models were generated based on the alignment of these pharmacophores. A five point pharmacophore with three hydrogen bond acceptors (A), two aromatic rings (R) as pharmacophore features was used to build the 3D-QSAR model with good statistical significance and predictive ability. Hypothesis AAARR.594 was observed more accurate than other models, with good *R*^2^ = 0.954, *Q*^2^ = 0.827. Additionally, visualization of the contour maps provided detail information of the relationship between structure and activity, and hence offer clues for designing more potent analogs. Further, the pharmacophore model was employed to screen against the database of natural product and synthetic compounds. The 672 hits retrieved during the study were evaluated using multiple criteria yielding 14 leads. Further investigation of lead molecules using ADME prediction suggested that their pharmacokinetic properties were found to be in an acceptable range.

## Additional files

10.1186/s40064-016-2589-3 3D-QSAR contour maps.
10.1186/s40064-016-2589-3 Statistical results of AAAHH.338 hypothesis and calculated activity of known β-1,3-glucan synthase inhibitors.
10.1186/s40064-016-2589-3 Detail of training and test set selection procedure.
