# Supplementary material for: In silico screening for identification of novel β-1,3-glucan synthase inhibitors using pharmacophore and 3D-QSAR methodologies
Source: Springerplus. 2016 Jul 4;5(1):965. doi: 10.1186/s40064-016-2589-3 (PMC4932017; doi:10.1186/s40064-016-2589-3)
Supplement: Supplementary file 1 — 10.1186/s40064-016-2589-3 3D-QSAR contour maps. [file 40064_2016_2589_MOESM1_ESM.docx]

**In silico screening for identification of novel β-1, 3-glucan synthase inhibitors using pharmacophore and 3D-QSAR methodologies**

**Potshangbam Angamba Meetei^a^, R .S. Rathore^b,c^, N Prakash Prabhu^a^, Vaibhav Vindal^a,b*^**

*^a^Department of Biotechnology and Bioinformatics*

*School of Life sciences, University of Hyderabad, Hyderabad 500046, India.*

*^b^Bioinformatics Infrastructure Facility,*

*School of Life sciences, University of Hyderabad, Hyderabad 500046, India.*

*^c^Centre for Biological Sciences, School of Earth, Biological and Environmental Sciences,*

*Central University of South Bihar, Patna 800014 India*

**^*^**corresponding authors [e-mail:-Vaibhav Vindal: vvls@uohyd.ernet.in]

Tel: +91-40-23134589

**Training and test set selection**

The selection of training and test set molecules is one of the key factors that influences a QSAR model predictive ability of unknown chemicals (Eriksson *et al.* 2003). Accurate prediction of the unknown chemical depends on the chemical space represented by the training set molecules. Consequently, the training and test sets must be divided in such a way to ensure maximum chemical space representation. Each representative point (multidimensional descriptor space) of test set must be close to those of the training set (Golbraikh and Tropsha 2000). There are many techniques available for separating the data, however, in this study, a non-hierarchical clustering method (*k*-means clustering) was employed (Macqueen 1967). The procedure involves clustering the series of compounds based on their chemical descriptors into a group of representative classes, according to number of *k* clusters. The procedure ensures maximum chemical space represention for both the training and test sets molecules.

For separating the pyridazionone derivatives into training and test sets, topological descriptors of the molecules were calculated using CDK tool (Steinbeck *et al*. 2003). Based on the topological descriptors, eight clusters were generated using *k*-means clustering technique. From each of the clusters, one representative molecule was selected to create the test set. From the clusters which have more molecules in a group (2, 5 and 8), two representative molecules were chosen (Table 1).

| Table 1. Clustering of the pyridazionone derivatives as training and test sets | |
| --- | --- |
| **Clusters** | **Molecules** |
| 1 | 7, 4, 16* |
| 2 | 2*, 13*, 15, 33, 34, 35 , 40 |
| 3 | 8, 17, 24*, 31, 32 |
| 4 | 5, 6*, 9, 10 |
| 5 | 27, 28, 29, 30*, 36*, 37, 38, 41 |
| 6 | 12, 18, 19, 25* |
| 7 | 1*, 3, 14, 22, 42 |
| 8 | 11, 20*, 21, 23, 26, 39* |

**References:**

Eriksson L, Jaworska J, Worth AP, et al (2003) Methods for Reliability and Uncertainty Assessment and for Applicability Evaluations of Classification- and Regression-Based QSARs. Environ Health Perspect 111:1361–1375. doi: 10.1289/ehp.5758

Golbraikh A, Tropsha A (2000) Predictive QSAR modeling based on diversity sampling of experimental datasets for the training and test set selection. Mol Divers 5:231–243.

Macqueen J (1967) Some Methods for classification and analysis of multivariate observations. University of California Press, pp 281–297

Steinbeck C, Han Y, Kuhn S, et al (2003) The Chemistry Development Kit (CDK):  An Open-Source Java Library for Chemo- and Bioinformatics. J Chem Inf Comput Sci 43:493–500. doi: 10.1021/ci025584y
