# Supplementary material for: In silico screening for identification of novel β-1,3-glucan synthase inhibitors using pharmacophore and 3D-QSAR methodologies
Source: Springerplus. 2016 Jul 4;5(1):965. doi: 10.1186/s40064-016-2589-3 (PMC4932017; doi:10.1186/s40064-016-2589-3)
Supplement: Supplementary file 2 — 10.1186/s40064-016-2589-3 Statistical results of AAAHH.338 hypothesis and calculated activity of known β-1,3-glucan synthase inhibitors. [file 40064_2016_2589_MOESM2_ESM.docx]

**In silico screening for identification of novel β-1, 3-glucan synthase inhibitors using pharmacophore and 3D-QSAR methodologies**

**Potshangbam Angamba Meetei^a^, R .S. Rathore^b,c^, N Prakash Prabhu^a^, Vaibhav Vindal^a,b*^**

*^a^Department of Biotechnology and Bioinformatics*

*School of Life sciences, University of Hyderabad, Hyderabad 500046, India.*

*^b^Bioinformatics Infrastructure Facility,*

*School of Life sciences, University of Hyderabad, Hyderabad 500046, India.*

*^c^Centre for Biological Sciences, School of Earth, Biological and Environmental Sciences,*

*Central University of South Bihar, Patna 800014 India*

**^*^**corresponding authors [e-mail:-Vaibhav Vindal: vvls@uohyd.ernet.in]

Tel: +91-40-23134589

| **Table S1.** Summary of PHASE 3D-QSAR statistical results low scoring AAAHH.338 hypothesis, the model is shown in bold font. | | | | | | | | | |
| --- | --- | --- | --- | --- | --- | --- | --- | --- | --- |
| **No. of Factors *^a^*** | **SD *^b^*** | **R-squared *^c^*** | **F *^d^*** | **P *^e^*** | **Stability *^f^*** | **RMSE *^g^*** | **Q-squared *^h^*** | **Pearson-R *^i^*** | ***R^2^_pred_ ^j^*** |
| 1 | 0.3751 | 0.617 | 51.5 | 3.76e-08 | 0.829 | 0.2226 | -0.2204 | 0.5247 | 0.366 |
| 2 | 0.2532 | 0.831 | 76.2 | 1.08e-12 | 0.6317 | 0.2170 | 0.3042 | 0.7605 | 0.387 |
| 3 | 0.145 | 0.8463 | 176.3 | 3.86e-19 | 0.5689 | 0.2015 | 0.4555 | 0.7573 | 0.398 |
| **4** | **0.1132** | **0.8684** | **222.1** | **2.66e-21** | **0.5177** | **0.1953** | **0.5952** | **0.8188** | **0.412** |

*^a^* Number of factor used in the analyses.

*^b^* (SD) the standard deviation of regression

*^c^* (*R*^2^) coefﬁcient of determination,

*^d^* (F) the ratio of the model variance to the observed activity variance

*^e^* (P) signiﬁcance level of F when treated as a ratio of Chi squared distributions

*^f^* ( Stability) stability of the model predictions to changes in the training set composition

*^g^* (RMSE ) the RMS error in the test set predictions

*^h^* (*Q*^2^) directly analogous to *R*^2^ but based on the test set predictions

*^i^*(Pearson-R) value for the correlation between the predicted and observed activity for the test set

*^j^*(*R^2^_pred_* ) Predictive *R^2^*, Standard deviation of error prediction.

| **Table S2**. Predicted biological activity of β-1,3-glucan synthase inhibitors using the AAARR.594 derived 3D-QSAR model | | | | |
| --- | --- | --- | --- | --- |
| S.NO | ZINC database ID | Compound (Synthetic) | Experimental  pIC50 | Predicted  pIC50 |
| 1 | Enfumafungin |  | 6 | 5.15 |
| 2 | Ergokonin A |  | 5.5 | 5.38 |
| 3 | Arundifungin |  | 5.2 | 5.26 |
| 4 | Ascosteroside |  | 5 | 5.4 |
