# Supplementary material for: In silico screening for identification of novel β-1,3-glucan synthase inhibitors using pharmacophore and 3D-QSAR methodologies
Source: Springerplus. 2016 Jul 4;5(1):965. doi: 10.1186/s40064-016-2589-3 (PMC4932017; doi:10.1186/s40064-016-2589-3)
Supplement: Supplementary file 3 — 10.1186/s40064-016-2589-3 Detail of training and test set selection procedure. [file 40064_2016_2589_MOESM3_ESM.docx]

**Additional Information**

**In silico screening for identification of novel β-1, 3-glucan synthase inhibitors using pharmacophore and 3D-QSAR methodologies**

**Potshangbam Angamba Meetei^a^, R .S. Rathore^b,c^, N Prakash Prabhu^a^, Vaibhav Vindal^a,b*^**

*^a^Department of Biotechnology and Bioinformatics*

*School of Life sciences, University of Hyderabad, Hyderabad 500046, India.*

*^b^Bioinformatics Infrastructure Facility,*

*School of Life sciences, University of Hyderabad, Hyderabad 500046, India.*

*^c^Centre for Biological Sciences, School of Earth, Biological and Environmental Sciences,*

*Central University of South Bihar, Patna 800014 India*

**^*^**corresponding authors [e-mail:-Vaibhav Vindal: vvls@uohyd.ernet.in]

Tel: +91-40-23134589


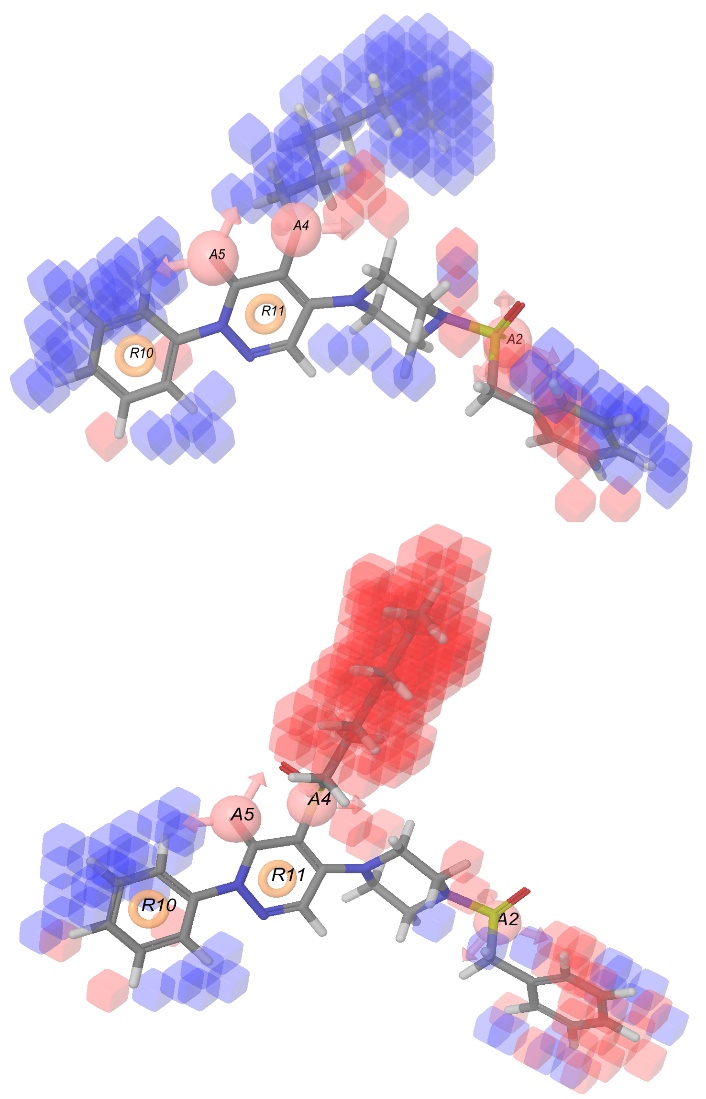

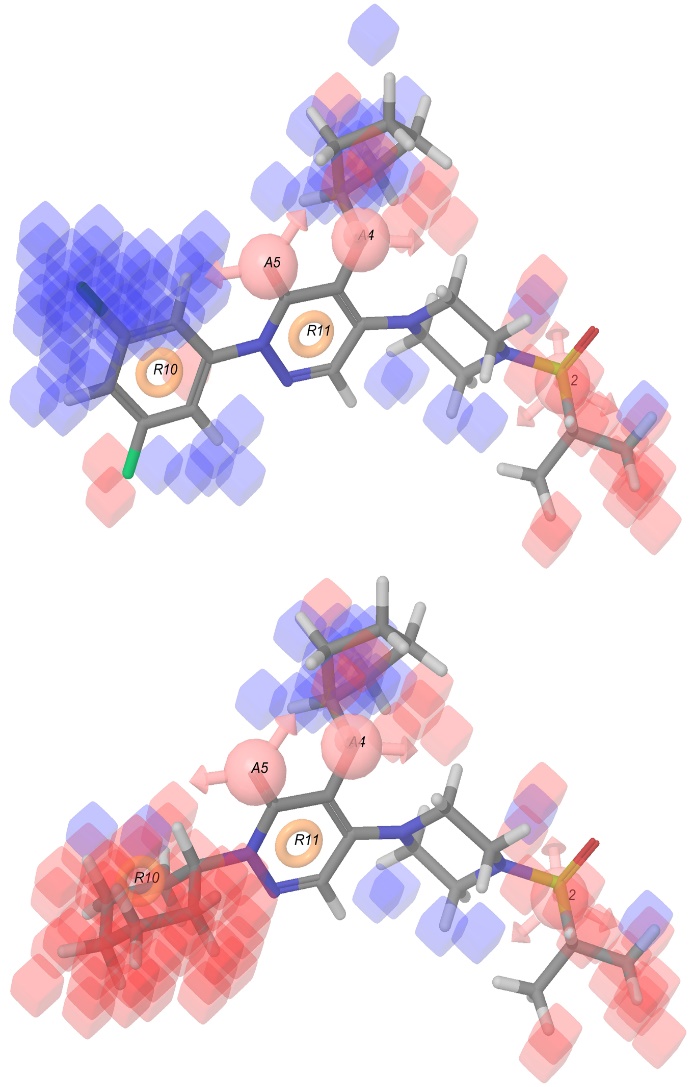


1. (B)


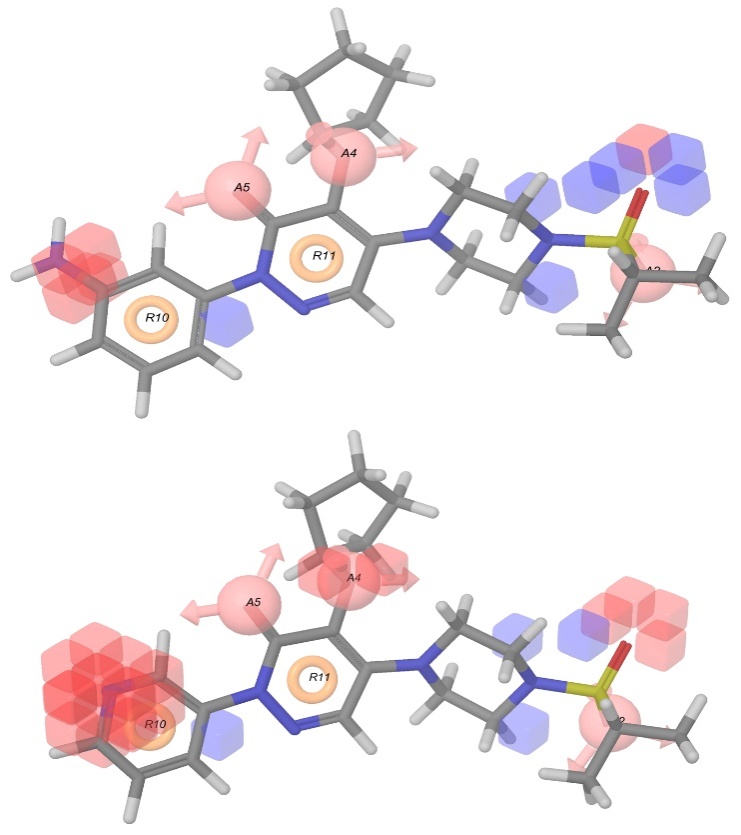


(C)

**Figure. S1** 3D-QSAR contour map in the context of (A) hydrophobic interactions represented on molecules **20** (upper) and **22** (lower) at the A4 pharmacophoric site ; (B) also on molecules **36** (upper) and **31** (lower) at the R10 pharmacophore feature position; (C) Electron withdrawing contours shown on molecules **39** (upper) and **32** (lower) at pharmacophoric position R10 ; blue cubes indicate favorable while red cubes unfavorable coefﬁcients; circles signifies the substitution position under consideration for the contour map analysis.
